# Potential microbial contamination during sampling of permafrost soil assessed by tracers

**DOI:** 10.1038/srep43338

**Published:** 2017-02-23

**Authors:** Toke Bang-Andreasen, Morten Schostag, Anders Priemé, Bo Elberling, Carsten S. Jacobsen

**Affiliations:** 1 Department of Environmental Science, Aarhus University, DK-4000 Roskilde, Denmark; 2 Department of Biology, University of Copenhagen, DK-2100 Copenhagen, Denmark; 3 Center for Permafrost (CENPERM), Department of Geosciences and Natural Resource Management, University of Copenhagen, DK-1350 Copenhagen, Denmark

## Abstract

Drilling and handling of permanently frozen soil cores without microbial contamination is of concern because contamination e.g. from the active layer above may lead to incorrect interpretation of results in experiments investigating potential and actual microbial activity in these low microbial biomass environments. Here, we present an example of how microbial contamination from active layer soil affected analysis of the potentially active microbial community in permafrost soil. We also present the development and use of two tracers: (1) fluorescent plastic microspheres and (2) *Pseudomonas putida* genetically tagged with Green Fluorescent Protein production to mimic potential microbial contamination of two permafrost cores. A protocol with special emphasis on avoiding microbial contamination was developed and employed to examine how far microbial contamination can penetrate into permafrost cores. The quantity of tracer elements decreased with depth into the permafrost cores, but the tracers were detected as far as 17 mm from the surface of the cores. The results emphasize that caution should be taken to avoid microbial contamination of permafrost cores and that the application of tracers represents a useful tool to assess penetration of potential microbial contamination into permafrost cores.

During the last two decades, permafrost and its microbial life have received increasing attention for several reasons: (1) Microorganisms are the main drivers in the conversion of permafrost organic matter into greenhouse gases^1^,^2^,^3^,^4^, and may have a marked impact on global climate if only a fraction of the stock of organic matter stored in permafrost is released as greenhouse gases to the atmosphere^5^,^6^. (2) Isolation, cultivation and characterization of microorganisms from permafrost may reveal novel microbial adaptations to e.g. low temperature and high osmolarity^7^,^8^ and may lead to the revival of ancient microorganisms^9^. (3) Permafrost is used as a model for extraterrestrial life^10^,^11^,^12^. (4) Ancient DNA extracted from the DNA-preserving permafrost environment is a unique window to the past^13^,^14^,^15^.

Permafrost samples are prone to contamination problems because they generally contain a low abundance of microorganisms compared to the overlaying active layer soils^16^,^17^,^18^. Microbial contamination of permafrost samples can result in false-positive and highly unreliable results when studying microbial functions and activity in permafrost, cultivating and isolating permafrost microorganisms, and isolating ancient DNA from permafrost. Contamination may especially compromise incubation studies of permafrost samples exposed to temperatures above 0 °C. Here, the introduction of just few exogenous microorganisms potentially better adapted to the incubation conditions compared to the permafrost bacteria may lead to a shift in the microbial community and, hence, the processes mediated by the microorganisms.

Permafrost soils are mainly sampled by drilling. Drilling and subsequent handling and processing of permafrost cores can introduce microbial contamination to the permafrost^19^,^20^. However, rarely studies report how microbial contamination was avoided^21^,^22^,^23^,^24^. The sources of microbial contamination in permafrost cores include the use of drilling liquid and improper handling of permafrost cores during post-drilling processes. The active layer soil has a larger abundance of microorganisms compared to the under-lying permafrost^16^,^17^ and drilling liquid may transfer microorganisms from the active layer to the permafrost where they may penetrate into the permafrost core. Transfer of microorganisms from the active layer may also happen when drilling liquid is not used because drilling itself produces heat which creates drilling mud of layers above the actual drilling spot. In both cases, sediment and ice may partly thaw and contaminate the sample which cannot be detected visually when the sample is recovered at the surface due to refreezing.

Methodological procedures have been applied during permafrost drilling and post-drilling processes to avoid microbial contamination including drilling without the use of drilling liquid^25^,^26^, aseptically scraping off the outer layers of permafrost cores in order to remove potential contamination within these layers^3^,^27^,^28^,^29^,^30^,^31^,^32^, using an exogenous bacterial tracer such as *Serratia marcescens*^27^,^31^,^33^,^34^,^35^, and using fluorescent microspheres and genetically marked strains to trace potential microbial contamination into permafrost cores^1^,^18^,^19^. A commonly used method to avoid contamination in permafrost cores is to scrape off the core’s exterior, often in combination with fluidless core drilling, but this method alone does not guarantee complete removal of microbial contamination. The use of fluorescent microspheres and genetically marked bacterial strains as tracers can indicate if microbial cells are able to penetrate into the permafrost core layers of interest and can be used both during drilling and post-drilling procedures^19^. Despite of this, tracers are rarely used as a tool to exclude microbial contamination of permafrost samples and knowledge about how far microbial contamination can reach into permafrost cores remain sparse.

In this study, we demonstrate how microbial contamination of permafrost soil cores can affect analysis of the potential activity of permafrost bacteria. Furthermore, we test two tracers consisting of fluorescent plastic microspheres and a bacterial strain carrying a green fluorescent protein (GFP) gene to estimate the potential for microbial contamination in permafrost cores. Tracer elements were quantified at increasing depths into two permafrost cores and showed how far microbial contamination can penetrate into permafrost cores. Additionally, we suggest a protocol to collect permafrost soil free of microbial contamination.

## Methods

### Permafrost cores

“Zackenberg” permafrost core (5 cm long, 10 cm diameter) was collected by fluidless drilling in August 2008. The core was obtained from a high Arctic wet grassland near Zackenberg Research Center, NE Greenland (74°30′N, 20°30′W) with continuous deposition of sediment and organic matter. The sample was obtained at a depth of 65–75 cm, which is just beneath the maximum thaw depth of the active layer in late August.

“Adventdalen 1” permafrost core (10 cm long, 5 cm diameter) was collected by fluidless drilling in high Arctic tundra, summer 2012, in Adventdalen, Svalbard, Norway (78°11′N, 15°55′E). The core was obtained from the upper part of the frozen permafrost at a depth of 80–120 cm, in a low-centered ice-wedge polygon. The sampling site is located at the outermost part of an alluvial fan, which is covered with Late Holocene loess deposits.

“Adventdalen 2” permafrost cores (11 cm long, 4.8 cm diameter) were collected on the 17^th^ of June, 22^nd^ of July and 2^nd^ of December 2010. The cores were obtained from a high Arctic heath in Adventdalen, Svalbard (78°10′N, 16°3′E), from a depth of 142–153 cm below ground surface. All cores were collected using a motorized fluidless hand drill with an expandable drill string and a drill head. Each core was pushed out of the drill head directly into a sterile plastic bag.

All cores were kept frozen and stored at −18 °C prior to further analysis. The “Adventdalen 2” permafrost cores were used to examine bacterial community structure, as described below, without much concern about possible microbial contamination. No tracers were added and no outer layers were removed from these cores. The “Zackenberg” and “Adventdalen 1” permafrost cores were used to test for potential microbial contamination and tracers were applied to these cores and outer layers were removed, as described below.

### Physical-chemical parameters

Grain size distribution was analyzed by dispersing permafrost sediment in 0.01 M tetrasodium pyrophosphate and demineralized H_2_O by 2 min of ultrasound and then analyzed by laser diffraction on a Mastersizer 2000 (Malvern Instruments Ltd, Malvern, UK). The pH-value was measured in soil slurries of 1:5 w/v ratio of soil to demineralized H_2_O using a PHM 80 pH-meter (Radiometer, Copenhagen, Denmark). Water/ice content was determined gravimetrically by drying a permafrost subsample to a constant mass at 105 °C. Total C and N were determined by combustion of dry soil at 1200 °C and 800 °C, respectively, followed by analyses on a TrueSpec CN determinator (LECO Corporation, St Joseph, MO, USA). Extracts were made for the analysis of dissolved organic carbon (DOC), nitrate (NO_3_^−^) and ammonium (NH_4_^+^) by shaking 1:5 w/v of sediment to demineralized H_2_O for 5 hours followed by centrifugation at 4300 RPM for 10 min and filtering the supernatant through GF/D filters (Whatman™, Maidstone, UK). The concentration of DOC was measured on the filtered soil extracts by first removing inorganic C by acidifying sample extracts with 2 M HCl, and then analyzing them on a TOC-5000A/SSM-5000A (Shimadzu, Kyoto, Japan). Concentrations of NO_3_^−^ and NH_4_^+^ were determined by flow injection analysis using a FIAstar™ 5000 (FOSS, Hillerød, Denmark) following the manufacturer′s instructions.

### DNA and RNA co-extraction, library preparation and bioinformatics

For the “Adventdalen 2” permafrost cores, DNA and RNA were co-extracted for analysis of 16 S rRNA genes and transcripts. DNA and RNA co-extraction and 16 S rRNA/rDNA library preparation were performed as previously described^36^ except that we used a two-step PCR protocol to get sufficient product for DNA/cDNA sequencing. The samples were first PCR amplified using the primers 515 F (5′-GTGCCAGCMGCCGCGGTAA-3′) and 806 R (5′-GGACTACHVGGGTWTCTAAT-3′)^37^ for 20 cycles. Then the PCR products were purified using carboxyl-coated magnetic beads (SPRI beads, Agencourt AMPure XP, Agencourt, Beverly, MA, USA), followed by 25 cycles of PCR using primers with Illumina adapters and barcodes and sequenced and analyzed as previously described^36^. The sequences have been deposited at the European Nucleotide Archive with the study accession number PRJEB15862.

### Tracer

The final tracers consisted of commercially available 0.5 μm carboxylated polystyrene yellow-green fluorescent microspheres (Fluoresbrite^®^ ; Polysciences Inc., Warrington, PA, USA) and a kanamycin resistant *Pseudomonas putida* strain with GFP gene insert^38^. The tracers were detected by both epi-fluorescent microscopy (targeting the microspheres) and real-time qPCR (targeting the GFP gene), as described later.

The *P. putida* tracer was propagated by growing the strain for 2 days on LB medium containing kanamycin (50 μg mL^−1^) at 20 °C. After 2 days of growth, the strain was washed in 0.015 M potassium phosphate buffer, pH 7.4, and incubated in the buffer overnight. The number of bacteria in the buffer was determined by Acridine Orange (AO) staining^39^. Briefly, 1 μL of bacteria-containing buffer was diluted in 5 ml sterile Milli-Q water and filtered through 0.2-μm polycarbonate filters (Nucleopore, Pleasanton, CA, USA). The filters were then placed floating on top of a 0.4% AO-solution for 2 min allowing AO to bind to the DNA and RNA of the bacteria. Excess AO was washed away by placing the filters on top of sterile Milli-Q water for 2 min and then drained of excess water. Finally, the filters were mounted on microscope slides followed by immediately counting the number of bacteria cells on the filter using an epifluorescence microscope (BX50 F; Olympus, Tokyo, Japan). The remaining bacteria-containing buffer was stored at −80 °C for later use in the final tracer.

The final combined tracer was produced on the day of use, by combining one volume undiluted (3.64 · 10^11^ microspheres ml^−1^) fluorescent microspheres with 199 volumes bacteria-containing buffer. The combined tracer was then washed three times with sterile water by centrifuging three times at 8000 rpm for 5 min, discarding of the supernatant and dissolving pellet in sterile water. The tracer solution was then added to sterile spray dispensers and ready to be used. The number of microspheres (1.27 ± 0.089 · 10^9^ microspheres mL^−1^; n = 10) and *P. putida* cells (6.47 ± 0.594 · 10^9^ cells mL^−1^; n = 10) in the final tracer was quantified by direct epi-fluorescent microscopy and AO staining, respectively.

### Tracer application

**Figure 1:**
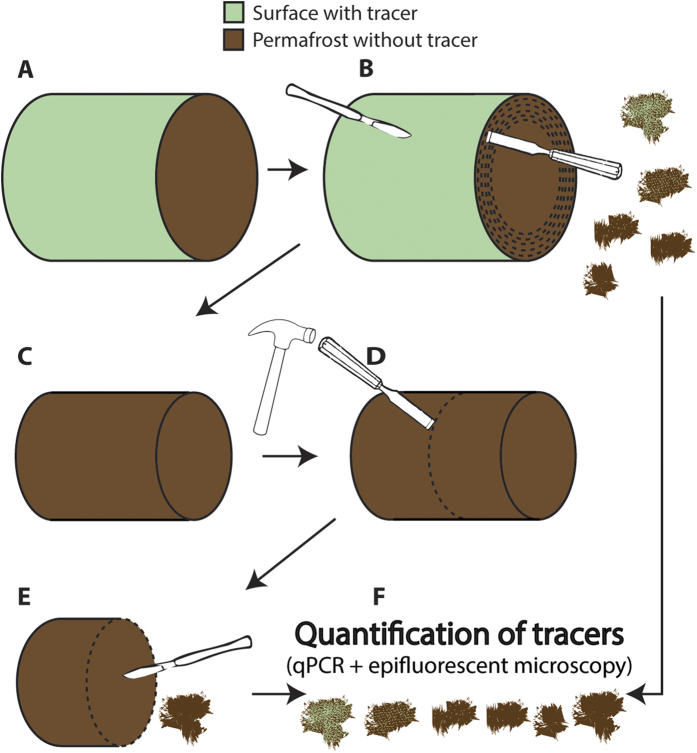
Procedure for tracer quantification: (**A**) Curved outer sides of the permafrost core was covered with tracers. (**B**) The outer layers (dashed lines) were removed one by one using sterile tools and extreme caution to avoid cross contamination between layers. (**C**) Resulting inner core, smaller in diameter and free of tracers and potential microbial contamination. (**D**) The inner core was transversely split in two using sterile chisel. (**E**) From the newly exposed surface, soil was collected from the center of the inner core for quantification of tracer elements. (**F**) Quantification of tracers in all the collected layers and from the center of the permafrost core.

The tracer solution was sprayed on the permafrost cores covering the curved surface of the frozen “Zackenberg” and “Adventdalen 1” cores (30 mL tracer solution on each core) (Fig. 1A). The cores were subsequently stored at −20 °C overnight. Processing was done inside a UV-radiation sterilized laminar airflow workbench in a −16 °C freeze-laboratory. The outer 21 mm of the “Zackenberg” core and the outer 20 mm of the “Adventdalen 1” core were cautiously scraped and chiseled off using sterile tools (Fig. 1B). This was done in six layers for the “Zackenberg” permafrost with depth intervals of [0–1], [1–5], [5–9], [9–13], [13–17] and [17–21] mm (Fig. 1B) plus soil from the center of the core (Fig. 1C–E) and in four layers for the “Adventdalen 1” permafrost with depth intervals of [0–5], [5–10], [10–15] and [15–20] mm (Fig. 1B). All soil layers were collected separately in sterile bags and homogenized by hammering on the outside of the sterile bags. Extreme caution was taken to avoid cross contamination between the different layers: All used surfaces in the freeze-laboratory were wiped in 70% DNase- and RNase-free ethanol and in sodium hypochloride (5% active chloride) prior to processing the permafrost cores and in between collection of the different permafrost layers. Nitrile gloves and sterile scalpels were used, along with autoclaved chisels and other autoclaved tools. New sterile tools and nitrile gloves were used when handling a new layer. In cases where tools had to be reused for sampling of different layers, the tools were thoroughly washed for 10 min in 70% DNase- and RNase-free ethanol followed by 10 min in sodium hypochloride (5% active chloride) before being used again.

### Detection of tracer

Detection and quantification of the fluorescent microspheres (Fig. 1F) were carried out on triplicates of 0.1 g permafrost soil from each layer. The triplicates were transferred to sterile 1.5-mL Eppendorf tubes and 300 μl sterile Milli-Q water were added and vortexed thoroughly. Thirty μL of this solution were transferred to a microscope slide and fluorescent microspheres were counted using epi-fluorescent microscopy. The enumeration was done on ten random places of the microscope slide using the microscope reticle grid (each 0.01 mm^2^). If no microspheres were detected in the first ten randomly chosen places, a screening for microspheres on the whole microscope slide was carried out (see Supplementary information S1 for more details).

Detection and quantification of GFP genes from the *P. putida* strain were carried out using qPCR on a CFX96™ Real-Time System, C1000 Touch™ Thermal Cycler (Bio-Rad, Richmond, VA, USA), with primers targeting the GFP gene: DNA was extracted from triplicates of 0.5 g freeze-dried permafrost soil (soil freeze-dried overnight using ScanVac CoolSafe (Labogene, Lynge, Denmark)) from each layer using the PowerLyzer™ PowerSoil^®^ DNA Isolation Kit (MoBio, Carlsbad, CA, USA). qPCR was performed on technical triplicates of the DNA extracts using a master mix consisting of 2 μl bovine serum albumin (Bioron, Ludwigshafen, Germany), 0.8 μL forward primer (gfp_f_; 5′-GCATGCGTAAAGGAGAAGAACTTTTCA-3′), 0.8 μL reverse primer (gfp_r_; 5′-AAGCTTATTTGTATAGTTCATCCATGC-3′)^40^, 10 μL Sibir master mix (Bioron), 1 μL DNA template and water to a total volume of 20 μL. PCR cycles were carried out with conditions slightly modified from a published protocol^40^: Initial denaturation and enzyme activation at 94 °C for 2 min followed by 50 cycles of denaturation at 94 °C for 45 sec, primer annealing at 60 °C for 30 sec, elongation at 72 °C for 1 min and 77.5 °C for 10 sec followed by measurement of relative fluorescence units (RFU) and finally one cycle of 72 °C for 6 min. After this program, melting curves for the PCR products were obtained to check for specificity of the PCR by performing a 0.5 °C stepwise increase in temperature from 64 °C to 95 °C with a RFU measurement after each step. The PCR efficiency for the assay was 88.9%, and R^2^ was 0.998. As GFP standards, we used DNA extracted from the dilution series made as a part of the GFP detection limit experiment, described next.

The qPCR detection limit of the tracer was determined by a dilution series (10^1^–10^9^) of the *P. putida-containing* buffer using 100-fold dilution steps. In triplicates, 50 μL of each dilution were added to 0.5 g of sand (particle size of half 50–70 mesh, half 20–30 mesh). Samples were then mixed thoroughly and incubated at 4 °C for 1 hour. DNA extraction and qPCR runs targeting the GFP gene of the samples were carried out as described above.

### Statistical analysis

One-way analysis of variance (ANOVA) was used to analyze for significant differences of the tracer elements with increasing depth into the permafrost. Paired student t-test was used to analyze for significant differences between summer and winter months of the mean Shannon-Weaver diversity index for the 16 S rRNA and rDNA based communities. RStudio version 0.99.484^41^ was used and a p-value < 0.05 was considered significant.

## Results

### Physical-chemical parameters

Physical-chemical parameters of the three permafrost cores are listed in Table 1.

### Bacterial community

**Figure 2:**
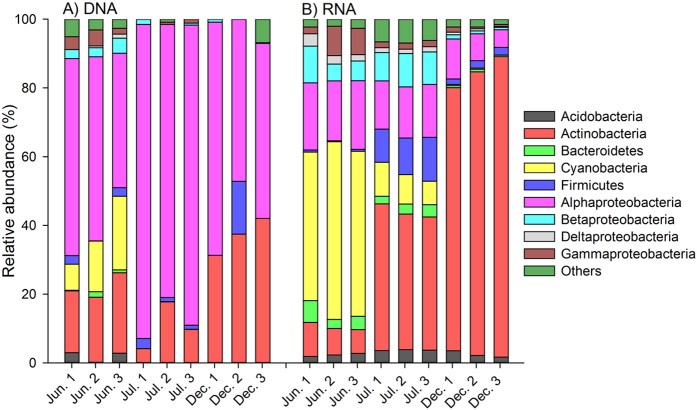
Relative abundance in June, July and December of bacterial phyla based on (**A**) 16 S rDNA and (**B**) 16 S rRNA in the 1.5 meter deep “Adventdalen 2” permafrost samples. The phylum Proteobacteria is separated into the four classes Alpha-, Beta-, Gamma- and Deltaproteobacteria. “Others” represents phyla with average relative abundance <1%. Replicates represent three DNA/RNA extractions from the same core.

Analysis of “Adventdalen 2” permafrost cores collected at 1.5 m depth revealed 204, 93 and 62 different operational taxonomic units (OTUs) at 97% sequence identity in the DNA samples from June, July and December, respectively. For RNA samples the numbers of OTUs were 560, 557 and 445 for June, July and December, respectively. A high relative abundance of Cyanobacteria was found in the June DNA samples with the most dominant OTU belonging to the order Streptophyta (9.93 ± 1.74%, average ± standard error of the mean). The abundance of this OTU in July and December DNA samples was 0.10 ± 0.01% and 0.04 ± 0.01%, respectively. In the RNA samples, Cyanobacteria had a high relative abundance in both June and July, with the most dominant OTU representing Cyanobacteria belonging to the genus *Nostoc*, with an average relative abundance of 40.96 ± 3.69%, 6.23 ± 0.99% and 0.11 ± 0.08% for June, July and December, respectively (Fig. 2). The Shannon-Weaver diversity index was not significantly different between summer and winter months (*P* = 0.65) for DNA samples with diversity calculated to be 2.67 ± 0.38, 0.87 ± 0.19 and 1.45 ± 0.29 for June, July and December, respectively. For RNA samples, the Shannon-Weaver diversity index was significantly different between summer and winter months (*P* = 0.04) with diversity 3.08 ± 0.19, 4.30 ± 0.03 and 2.58 ± 0.16 for June, July and December, respectively.

### Tracer

**Figure 3:**
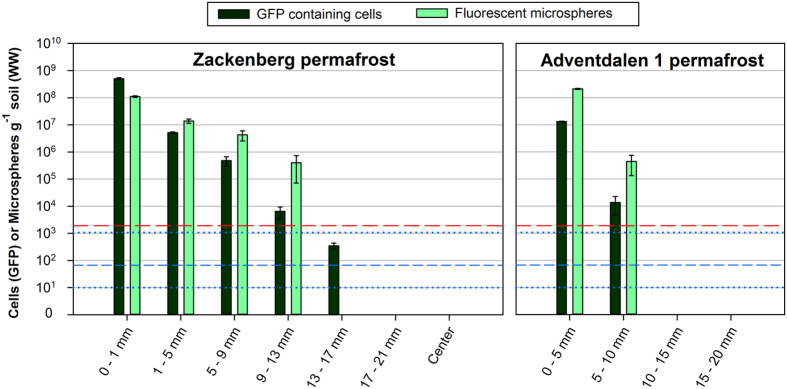
Number of tracer elements at different distances from the surface of the “Zackenberg” and “Adventdalen 1” permafrost cores. Red dashed line (long dash) and blue dashed line (short dash) indicate calculated detection limit for the fluorescent microspheres and the GFP containing cells, respectively. Blue dotted lines indicate the interval of the experimentally determined detection limit of the *P. putida*, GFP, tracer. Bars represent means ± standard error of the mean from nine replicates for each GFP measurement and 30 replicates for quantification of microspheres.

The quantity of both tracers (fluorescent microspheres and GFP-gene containing *P. putida*) decreased significantly (*P* < 0.001) with increasing depth into both permafrost cores, and tracer elements were found as far as 13–17 mm into the “Zackenberg” permafrost and 5–10 mm into the “Adventdalen 1” permafrost (Fig. 3).

The detection limit of fluorescent tracers was calculated as 1900 microspheres g^−1^ soil and 67 GFP containing cells g^−1^ soil (Fig. 3) (see Supplementary information S1 for detection limit calculations). This is consistent with the experimentally determined detection limit of the GFP gene of the *P. putida* tracer determined to be ranging from 10 to 1075 cells g^−1^ soil (Fig. 3).

## Discussion

The analysis of the “Adventdalen 2” permafrost cores drilled and processed without the use of tracers revealed that a large proportion of the potentially active bacteria at 1.5 m soil depth belonged to bacteria dependent on sunlight. Thus, the samples were shown to contain high relative abundance of an OTU belonging to the cyanobacterial genus *Nostoc* in the summer months (40.96% in June and 6.23% in July). This OTU was also detected in the active layer (top 10 cm below soil surface) of the core from the same sampling campaign, where the relative abundance of *Nostoc* at RNA level was 22.0% and 0.15% for the June and July samples, respectively^36^. *Nostoc spp.* and other Cyanobacteria are known to inhabit soil surfaces in Svalbard^42^,^43^. We consider the large relative abundance of potential active (RNA level) cyanobacteria in the permafrost summer samples as a strong indication of contamination, because sunlight is absent in permafrost 142–158 cm below the soil surface and Cyanobacteria need sunlight to be active at the level observed here. Furthermore, the Shannon-Weaver diversity significantly differed between summer and winter for the active bacterial community (RNA samples), with summer having a higher diversity. Since permafrost remain frozen and relatively stable over the year bacterial diversity is not expected to change much between summer and winter.

The microbial contamination in the “Adventdalen 2” permafrost cores was most likely a result of the core drilling procedure where two potential routes are known: 1) microbial contamination from upper soil layers (active layer) of the drilling hole via the drill-head, and 2) water or mud from the wet soil surface in summer washed into the drilling hole.

Cyanobacteria present in the DNA pool is not a clear indication of drill contamination of the permafrost core, since they have been detected in permafrost samples^44^ and viable cells have been isolated from permafrost samples^45^. Therefore, if only DNA results were present we would have no strong indications on microbial contamination of the samples.

These results show that caution should be taken to avoid microbial contamination when drilling and handling permafrost samples. They also show that microbial contamination can be difficult to detect on DNA level in bacterial community analysis of permafrost systems. Based on these findings we decided to develop, use and test tracers to mimic potential microbial contamination of permafrost cores and to develop a procedure to avoid microbial contamination in future studies, as discussed and described below.

Using fluorescent microsphere and GFP-based tracers, we were able to determine how far microbial contamination may penetrate into permafrost cores and thereby estimate how much of the core′s exterior needs to be removed to ensure uncontaminated permafrost samples. Permafrost soil collected deeper inside the cores, than tracer elements were detected, was considered uncontaminated and suitable for further investigations of permafrost microbiology. These results are consistent with findings of previous studies where exogenous tracer elements were not found in the center of permafrost cores^19^,^27^,^33^. However, we detected tracer elements as far as 13–17 mm into the “Zackenberg” permafrost, which, to our knowledge, is the furthest into a permafrost core potential contamination has been reported.

The tracers seemed to successfully mimic the transport of contaminating microorganisms into permafrost cores with different physical-chemical parameters and the quantity of tracer elements significantly decreased with depth into the cores. Soil structure and geochemistry determine how far into a permafrost core microbial contamination will penetrate. Particle size, ice content, concentration of humic substances and other soil parameters strongly influence size and numbers of pores and cavities in permafrost soil, and to what extent microorganisms are adsorbed to the soil particles, thus influencing how easily microbes penetrate into the core^46^. The tracer was found to penetrate deeper into the “Zackenberg” permafrost than into the “Adventdalen 1” permafrost. This is considered mainly due to the higher ice content of the “Adventdalen 1” permafrost and despite of a generally coarse grain size distribution and a higher porosity of the “Adventdalen 1” permafrost as compared to the “Zackenberg” permafrost. The ice content is present in the cavities and pores of the permafrost and acts as an effective physical barrier for the tracer and thereby also for microbial contamination. A practical concern arises with potential contamination as far as 17 mm into a permafrost core, namely the diameter of the drilling bit. A substantial part of the core exterior may need to be discarded to obtain uncontaminated permafrost and the diameter of the used drill bit therefore has to be large enough to obtain a considerable amount of uncontaminated permafrost.

In the “Zackenberg” permafrost core, GFP-coding genes were detected deeper into the core than the fluorescent microspheres, which most likely is the result of the higher sensitivity of the qPCR detection method compared to the epi-fluorescent microscopy detection method, as described in Supplementary information S1. Additionally, the 0.4–0.6 μm diameter of the *Pseudomonas putida* strain^47^ is comparable to the 0.5 μm spherical microspheres, but the bacterium may, due to its potentially smaller diameter, penetrate into slightly smaller pores and cavities in the permafrost, and thereby potentially deeper into the permafrost core, than the microspheres.

The tracers were applied to the permafrost cores post-drilling, but permafrost cores are exposed to microbial contamination during drilling, as contamination from upper soil layers of the soil profile can be transported to lower layers by the drilling equipment, thereby contaminating permafrost cores. Furthermore, heat is generated during drilling, especially when the drill penetrates a rock, which may thaw part of the permafrost core exterior allowing microbial contamination to reach further into the core. For this reason, drilling should be done at reduced speed and without the use of drilling fluids, and a tracer should be applied directly onto drilling bits before drilling^19^. Gene manipulated microbial tracers, such as the *P. putida* strain described in the present study, cannot be used in field but the fluorescent microspheres can. Permafrost samples are also exposed to exogenous microorganisms during post-drilling procedures and a tracer should preferably also be applied to permafrost cores in order to estimate the extent of contamination introduced here. Genetically modified microbial tracers can only be applied to permafrost cores under the right laboratory conditions and hence are only suitable for detecting potential microbial contamination introduced during laboratory work. These tracers act as a complementary detection method (qPCR/PCR) to the epi-fluorescent microscopy detection of fluorescent microspheres allowing for a very thorough examination of potential microbial contamination of permafrost cores.

The tracer application and procedure to avoid microbial contamination in permafrost cores described here are based on previously published literature that detect and quantify fluorescent microspheres^1^,^18^,^19^. Juck *et al*.^19^ quantified fluorescent microspheres up to 6 mm into a permafrost core and tested for presence or absence of GFP gene using PCR in exterior and interior parts of permafrost cores. The present study quantifies not only fluorescent microspheres using fluorescent microscopy, but also GFP genes using qPCR. The quantitative PCR detects contamination in layers deeper into permafrost cores than found with endpoint PCR by Juck *et al*.^19^. Together this strongly improves our knowledge about penetration of potential microbial contamination into permafrost cores.

**Figure 4:**
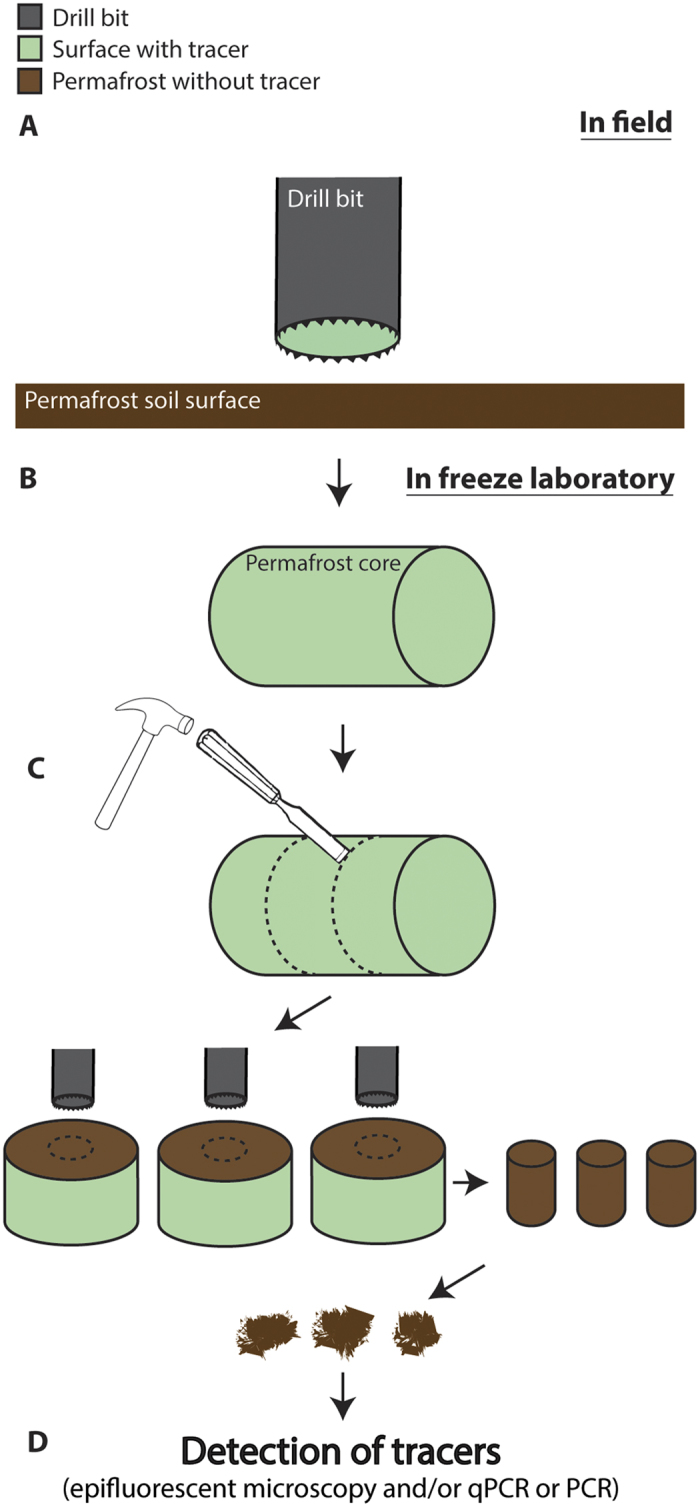
Recommended procedure to sample uncontaminated permafrost using tracers. In field: (**A**) Cover the permafrost cores with fluorescent microspheres as a tracer immediately after being collected or apply fluorescent microspheres as a tracer to drilling bits as described in published litterature^19^. Use fluidless drilling at reduced speed to minimize heat generation and transfer of microbial contamination. Store core at sub-zero conditions immediately after drilling. In freeze laboratory (work aseptically): (**B**) If not already done in field, cover the surface of the permafrost core with a tracer solution consisting of fluorescent microspheres and /or microbial strain with GFP insert (or other strain with genes not found in soil systems, which can be specifically detected using primers) and store core in freezer overnight. (**C**) In freeze laboratory split core transversely in desired number of sections using sterile chisels and collect permafrost samples from the center of the core at the newly exposed surface using hollow sterile drill bits or similar sterile tools. Collect samples in sterile bags. Be very cautious to not cross contaminate the center of the core with soil from outer layers of the core. Sterile foil is very useful at this step. (**D**) Detect tracer elements in the permafrost samples collected from the center of the core (and from the outer layer as positive control) using epi-fluorescent microscopy (detection of fluorescent microspheres) and qPCR - or regular PCR followed by gel-electrophoresis – using specific primers targeting the gene(s) from the microbial tracer. If no tracer elements are detected from the center of the core the sample collected from the center is considered uncontaminated permafrost.

Based on earlier studies and results from the present study, we propose a general procedure using tracers to ensure uncontaminated permafrost samples, described in Fig. 4. It is important to emphasize that processing of permafrost cores should be done aseptically. This is not possible during drilling (Fig. 4A), but post-drilling work on permafrost cores (Fig. 4B,C) should be carried out using sterile tools in a sterile laminar air flow bench, which has been exposed to UV radiation to kill microorganisms and destroy DNA and RNA. Other studies describe additional precautions like the use of body suit, face mask, cap and how to sterilize tools used for processing of permafrost cores^20^,^27^. qPCR is recommended as the molecular detection procedure of the tracer gene because of the low detection limit of this method. Alternatively, ordinary PCR followed by gel-electrophoresis-based visualization of amplified tracer gene can be performed in laboratories without access to a qPCR machine. This will provide information on presence or absence of the tracer gene but with a higher detection limit than if qPCR is used.

Other studies suggest an aseptical removal of the outer layers of permafrost cores sometimes in combination with application of bacterial tracer followed by sampling of the inner uncontaminated part of the core^3^,^27^,^28^,^29^,^30^,^31^. The scraping method allows to successfully collect uncontaminated permafrost but the protocol presented in this study has the advantages of circumventing the challenging scraping procedure where microbial contamination occasionally gets dragged into deeper permafrost layers by scraping tools. Using a chisel to split the permafrost core as described here the core is split in two in a way that prevents the chisel to transfer material from the outer permafrost core layers into the inner parts. The newly exposed surface can then be used to sample uncontaminated permafrost from the center of permafrost cores (Fig. 4C).

In summary, after detecting microbial contamination in permafrost core samples, we successfully developed and applied a post-drilling protocol involving two tracers mimicking penetration of microbial contamination into permafrost cores with different physical-chemical parameters. Our results indicate that the ice content of permafrost cores is the main barrier for the penetration of microbial contamination into permafrost cores with higher ice content resulting in less penetration of microbial contamination into permafrost cores. Tracer elements significantly decreased with increasing depth into permafrost cores and were detected as far as 17 mm into the permafrost core, which is the furthest potential contamination reported. Soil collected deeper into the permafrost cores, than the tracer was detected, was considered uncontaminated. The procedure described here is relatively easy to carry out and we suggest that it is applied to all experiments involving microorganisms, microbial processes and nucleic acids from permafrost cores. The procedure may also be applied to collection of subsurface ice core samples^19^,^48^.

## Additional Information

**How to cite this article**: Bang-Andreasen, T. *et al*. Potential microbial contamination during sampling of permafrost soil assessed by tracers. *Sci. Rep.* **7**, 43338; doi: 10.1038/srep43338 (2017).

**Publisher's note:** Springer Nature remains neutral with regard to jurisdictional claims in published maps and institutional affiliations.

## Supplementary Material

Supplementary Information

## Figures and Tables

**Table 1:** Physical-chemical parameters of the permafrost cores.

	Zackenberg	Adventdalen 1	Adventdalen 2	
pH	6.7	5.1	6.8	
Total C (%)	2.4 ± 0.001	4.5 ± 0.061	2.24	
Total N (%)	0.16 ± 0.013	0.33 ± 0.004	0.14	
DOC (mg L^−1^)	16.0 ± 0.67	12.7 ± 0.24	60.3	
NH^4+^ (mg L^−1^)	18.9 ± 6.4	1.5 ± 0.42	5.1	
NO_3_^−^ (mg L^−1^)	46.3 ± 0.4	0.11 ± 0.021	0.202	
Gravimetric water	(ice) content (%)	27.4	59.5	
Soil texture (% of clay (<2 μm); silt (2–63 μm); sand (>63 μm))	7.6; 74.4; 18.0	5.5; 47.6; 46.9	4.1; 68.4; 27.5	

The ± symbols indicate standard error of the mean, *n* = 3. Numbers without ± are measurements on a single replicate, n = 1, because too little soil was obtained to perform measurements on triplicates.
