# Erratum: Potential microbial contamination during sampling of permafrost soil assessed by tracers

**DOI:** 10.1038/srep46006

**Published:** 2017-04-06

**Authors:** Toke Bang-Andreasen, Morten Schostag, Anders Priemé, Bo Elberling, Carsten S. Jacobsen

Scientific Reports 7: Article number: 4333810.1038/srep43338; published online: 02 23 2017; updated: 04 06 2017

The original HTML version of this Article contained formatting errors in the “Gravimetric water (ice) content (%)” row. The correct Table appears below.

This error has been corrected in the HTML version of the Article; the PDF version was correct at the time of publication.

## Figures and Tables

**Table 1:** Physical-chemical parameters of the permafrost cores.

	Zackenberg	Adventdalen 1	Adventdalen 2
pH	6.7	5.1	6.8
Total C (%)	2.4 ± 0.001	4.5 ± 0.061	2.24
Total N (%)	0.16 ± 0.013	0.33 ± 0.004	0.14
DOC (mg L^−1^)	16.0 ± 0.67	12.7 ± 0.24	60.3
NH^4+^ (mg L^−1^)	18.9 ± 6.4	1.5 ± 0.42	5.1
NO^3−^ (mg L^−1^)	46.3 ± 0.4	0.11 ± 0.021	0.202
Gravimetric water (ice) content (%)	27.4	44.1	59.5
Soil texture (% of clay (<2 μm); silt (2–63 μm); sand (>63 μm))	7.6; 74.4; 18.0	5.5; 47.6; 46.9	4.1; 68.4; 27.5

*The* ± *symbols indicate standard error of the mean, n* = *3. Numbers without* ± *are measurements on a single replicate, n* = *1, because too little soil was obtained to perform measurements on triplicates.*

