# Supplementary material for: Potential microbial contamination during sampling of permafrost soil assessed by tracers
Source: Sci Rep. 2017 Feb 23;7:43338. doi: 10.1038/srep43338 (PMC5322388; doi:10.1038/srep43338)
Supplement: Supplementary Information [file srep43338-s1.pdf]

Supplementary information

Potential microbial contamination during sampling of permafrost soil assessed by tracers

Toke Bang-Andreasen[a,b,c], Morten Schostag[b,c], Anders Priemé[b,c], Bo Elberling[c], Carsten S. Jacobsen[a,c*]

[a] Department of Environmental Science, Aarhus University, DK-4000 Roskilde, Denmark.
[b] Department of Biology, University of Copenhagen, DK-2100 Copenhagen, Denmark.
[c] Center for Permafrost (CENPERM), Department of Geosciences and Natural Resource Management, University of Copenhagen, DK-1350 Copenhagen, Denmark.

*Corresponding author. Email: csj@envs.au.dk

Supplementary information S1: Detection limit of tracers

Epi-fluorescent microscopy

Detection and counting of fluorescent microspheres were done using a microscope reticle grid on an epi-fluorescent microscope at 100 X magnification. The reticle grid area at 100 X magnification was 0.01 mm^2 and the area of the coverslips containing the samples was 576 mm^2. The number of reticle grids on one coverslip was thereby:

$$\frac{576\ mm^2\ (coverslip\ area)}{0{,}01\ mm^2\ (microscope\ reticle\ grid\ area\ at\ 100\ X\ magnification)} = 57{,}600\ reticle\ grids \cdot coverslip^{-1}$$

For each microscopy sample, 10 minutes were used for detection of fluorescent microspheres. During this time period approximately 1000 reticle grids were inspected by counting fluorescent microspheres in the first ten randomly chosen reticle grids on the coverslip followed by fast manual screening for fluorescent microspheres if none were found in the first ten randomly chosen reticle grids. A single fluorescent microsphere in any of the 1000 reticle grids will be detected because the fluorescent microspheres fluoresce very strongly and can easily be detected apart from other fluorescent matter in the sample.

The coverslip contained 30 µl sample of a 300-µl solution of 0.1 g permafrost soil and technical triplicates were made for each sample. The epi-fluorescent microscopy detection limit was thereby:

$$\frac{1\ microsphere}{\frac{1{,}000\ grids\ screened \cdot coverslip^{-1}}{57{,}600\ grids \cdot coverslip^{-1}} \cdot \frac{30\ \mu l\ used \cdot 3\ tech.replicates}{300\ \mu l\ total\ volume} \cdot 0.1\ g\ soil} = \mathbf{1,900\ miscospheres \cdot g^{-1}\ soil}$$

qPCR

We assume that DNA from a single GFP gene containing cell in a qPCR-plate well can be detected by qPCR. For each well of the qPCR-plate 1 µl of a total of 100 µl of DNA extract was used from a 0.5 g permafrost soil sample and technical triplicates were made for each sample. The qPCR detection limit was thereby:

$$\frac{1\ GFP\ containing\ cell}{\frac{1\ \mu l\ DNA\ extract \cdot 3\ tech.replicates}{100\ \mu l\ total\ DNA\ extract} \cdot 0.5\ g\ soil} = 66.67 \approx \mathbf{67\ GFP\ gene\ containing\ cells \cdot g^{-1}\ soil}$$

This detection limit was in accordance with the experimentally obtained detection limit, which was determined to be 10 - 1075 GFP containing cells g^{-1} soil, as described in the Materials and Methods section.
